# Dry gel spinning of fungal hydrogels for the development of renewable yarns from food waste

**DOI:** 10.1186/s40694-024-00178-1

**Published:** 2024-08-02

**Authors:** Alice Lindh, E. R. Kanishka B. Wijayarathna, Göksu Cinar Ciftci, Samira Syed, Tariq Bashir, Nawar Kadi, Akram Zamani

**Affiliations:** 1 Swedish Centre for Resource Recovery, University of Borås, Borås, SE-501 90 Sweden; 2 RISE Research Institutes of Sweden, Stockholm, SE-114 28 Sweden; 3 Polymeric E-textiles, The Swedish School of Textiles, University of Borås, Borås, SE-50190 Sweden; 4 Department of Textile Technology, Faculty of Textiles, Engineering and Business, University of Borås, Borås, SE-50190 Sweden

**Keywords:** Food waste, Filamentous fungi, Rhizopus delemar, Fungal hydrogels, Dry gel spinning, Textile applications

## Abstract

**Background:**

Renewable materials made using environmentally friendly processes are in high demand as a solution to reduce the pollution created by the fashion industry. In recent years, there has been a growing trend in research on renewable materials focused on bio-based materials derived from fungi.

**Results:**

Recently, fungal cell wall material of a chitosan producing fungus has been wet spun to monofilaments. This paper presents a modification for the fungal monofilament spinning process, by the development of a benign method, dry gel spinning, to produce continuous monofilaments and twisted multifilament yarns, from fungal cell wall, that can be used in textile applications. The fungal biomass of *Rhizopus delemar*, grown using bread waste as a substrate, was subjected to alkali treatment with a dilute sodium hydroxide solution to isolate alkali-insoluble material (AIM), which mainly consists of the fungal cell wall. The treatment of AIM with dilute lactic acid resulted in hydrogel formation. The morphology of the hydrogels was pH dependent, and they exhibited shear thinning viscoelastic behavior. Dry gel spinning of the fungal hydrogels was first conducted using a simple lab-scale syringe pump to inject the hydrogels through a needle to form a monofilament, which was directly placed on a rotating receiver and left to dry at room temperature. The resulting monofilament was used to make twisted multifilament yarns. The process was then improved by incorporating a heated chamber for the quicker drying of the monofilaments (at 30⁰C). Finally, the spinning process was scaled up using a twin-screw microcompounder instead of the syringe pump. The monofilaments were several meters long and reached a tensile strength of 63 MPa with a % elongation at break of 14. When spinning was performed in the heated chamber, the tensile strength increased to 80 MPa and further increased to 103 MPa when a micro-compounder was used for spinning.

**Conclusion:**

The developed dry gel spinning method shows promising results in scalability and demonstrates the potential for renewable material production using fungi. This novel approach produces materials with mechanical properties comparable to those of conventional textile fibers.

**Supplementary Information:**

The online version contains supplementary material available at 10.1186/s40694-024-00178-1.

## Background

Global food waste has reached an alarming level in recent years, with the Food and Agricultural Organization of the United Nations (FAO) reporting an estimated 931 million tons per year [1]. Bread is one of the most common food waste categories in Sweden. According to a life cycle assessment (LCA) research, the amount of bread wasted per year in Sweden was estimated to be 80 410 tons corresponding to 8.1 kg of bread per person/year [2, 3]. Additionally, since bread is considered a nutritional substrate for microorganisms, it has been studied for the production of bioethanol, alternative food, feed, and even biomaterials through biotechnological processes [4–10].

The textile industry has the second-largest impact on the environment after the oil industry [11]. This contributes to a high demand for water and pesticides, especially during cotton farming, followed by dyeing and preparation steps to treat natural and synthetic fibers, filaments, and textiles for further applications [12]. Synthetic textile materials from fossil sources affect marine life by allowing microplastics to enter the sea [13]. The degradation time of synthetic polymers is difficult to estimate, but may take from 30 to 1000 years, which makes these textile materials a problem for a sustainable future [14].

In recent years, bread waste has been valorized into fungal products, such as monofilament textile fibers [8, 15, 16], bioplastic films [6], and leather-like materials [9]. In these studies, the *Mucoromycetes* fungus *Rhizopus delemar* (categorized as *Zygomycetes* in old taxonomic groups) cultivated in bread waste in a submerged cultivation process was used to develop different end products. The cell wall of the *Mucoromycotes* has a branched microfibrillar structure containing chitin and chitosan [17, 18]. Fungal cell wall should be isolated from fungal biomass for the production of fungal monofilaments [16]. The development of fungal textiles from bread waste is an environmentally friendly solution for both food waste and textile pollution.

Svensson, Ferreira [8], Svensson, Oliveira [15] and Wijayarathna, Mohammadkhani [16] have reported development of fungal monofilaments through wet spinning. The spinning dope was a hydrogel made from fungal cell wall of *Rhizopus delemar*. In brief, fungal cell wall material was isolated from the fungal biomass through an alkali treatment which dissolved and removed the proteins and other alkali soluble ingredients. This resulted in the recovery of cell wall material as alkali-insoluble material (AIM). The hydrogel was then prepared by adding lactic acid to AIM, which promoted the protonation of the amino groups of fungal chitosan and resulted in hydrogel formation. Injection of the fungal hydrogel into a coagulation bath containing ethanol led to simultaneous orientation of the fungal microfibers along the injection axis and water removal from the fungal hydrogel in the coagulation bath. This resulted in the formation of fungal monofilaments.

Generally, the use of coagulation agents and solvents in wet-spinning processes, requires nearly complete recovery to reduce the cost of the process [19]. In contrast, the dry gel spinning method, in which filaments are spun directly without the need for a coagulation agent, is considered a low-cost and environmentally friendly method. Having a shear thinning hydrogel as the spinning dope is important for both wet and dry gel spinning. Owing to the quick dehydration in ethanol coagulation bath wet spun monofilaments are brittle in nature. Due to the simple process of spinning, collecting, and drying longer monofilaments could be easily made with dry-gel spinning compared to wet-spinning. Dry spinning has been successfully tested for the spinning of monofilaments from nanocellulose hydrogels [20, 21].

The aim of the current study was to investigate the possibility of monofilaments spinning from fungal hydrogel through dry gel spinning. Bread waste was used to cultivate *Rhizopus delemar* in a pilot-scale bioreactor. The cell wall was isolated from fungal biomass and used for the preparation of fungal hydrogels. The fungal hydrogels were characterized in terms of morphology and rheological properties. The dry gel spinning of the hydrogels was performed using a small-scale custom-made setup, and the process was then scaled up. The produced filaments were characterized in terms of mechanical and thermal properties, as well as morphology. Development of the novel dry-gel spinning method for production of fungal monofilament not only improved the whole process in terms of reducing chemical usage but also improved scalability.

## Methods

### Chemicals and materials

Waste classified bread was obtained from ICA city supermarkets in Borås, Sweden. Sodium hydroxide, peptone, agar, α-amylase from *Bacillus licheniformis* (heat-stable), and L-(+)-lactic acid (88–92%) were purchased from Sigma Aldrich (Germany). Ethanol (absolute) was purchased from VWR (France). Anhydrous glucose was purchased from Fisher Scientific (Germany). *Rhizopus delemar* CBS 145,940 a bio safety level 1 (BSL-1) fungal strain (https://wi.knaw.nl/page/fungal_display/92976) was obtained from Westerdijk Fungal Biodiversity Institute (earlier known as Centraalbureau voor Schimmelcultures), Ultrecht, The Netherlands. Liquid nitrogen was purchased from Jerngruppen in Borås, Sweden.

### Pretreatment of bread

Bread obtained from ICA city Borås was dried at room temperature and milled into a powder (size < 3 mm). The milled bread was mixed with water into a 20 wt% mixture and heated to 80 °C in a 60 L kettle (Digiboil 65 L, KegLand, Australia). The pH was adjusted to 7 using 5.0 M NaOH [22]. Afterwards, 1.35 mL α-amylase from *Bacillus licheniformis* (heat stable, 20,000–60,000 Un/ml, Sigma Aldrich) was added to the mixture to initiate the starch liquefaction process. The liquefaction process was continued at 80 ℃ for 2 h [22]. The suspension was manually mixed every 15 min. Finally, the suspension was filtered through a 200-micron polyester filter bag (Brew bag^®^, USA), where the liquid fraction was separated from the insoluble remaining solids that were discarded. The dry weight content of the collected liquid was measured by drying triplicate samples at 70 °C for 24 h. This liquid fraction, which contained approximately 14 wt % solids, was used for fungal cultivation.

### Cultivation

The cultivation of *Rhizopus delemar* on bread waste was performed in three steps. First, the fungus was grown in petri dishes with agar medium (20 g/L glucose, 17.0 g/L agar, 4 g/L peptone) for 3 days at 30 ℃. As the inoculum for the next step, a spore suspension was made by adding 20 mL of sterile water to each plate and releasing the spores using a sterile L-shape plastic spreader. Two 500 mL Erlenmeyer flasks with 200 ml medium were prepared for the pre-culture containing the substrate diluted to 4% using water supplemented with yeast extract 1 g/L. The flasks were sterilized in an autoclave (VX95, Systec, Germany) for 20 min at 121 ℃. As the inoculum, 4 ml of spore suspension of *R. delemar* was added into each e-flask, and cultivation was performed in a water bath shaker (Grant Instruments Ltd, UK) for 24 h at 35 °C and 110 rpm.

Second, the cultivation process was scaled up to a 26 L bubble column bioreactor (Bioengineering, Switzerland) with 20 L of medium as the working volume. The empty reactor was sterilized (in situ) with steam for 20 min at 121 ℃. The substrate was diluted to 4 wt% and supplemented with 1 g/L yeast extract. The pH of the substrate was adjusted to 5.5, and it was sterilized in an autoclave (VX95, Systec, Germany) for 20 min at 121 °C. After cooling, it was mixed with the prepared pre-culture from the shake flasks and transferred to the reactor under aseptic conditions. The fungal cultivation was performed for 24 h at 35 ℃ in the 26 L reactor. The obtained cultivation broth was used as inoculum for the next step of cultivation in a 1.3 m^3^ bubble column bioreactor (Knislinge Mekaniska Verkstad AB, Sweden).

For the final step, 1000 L of the substrate was diluted to 4% and supplemented with 1 g/L yeast extract, pumped into the 1.3 m^3^ bioreactor where it was sterilized for 1 h at 80 °C. After cooling, the inoculum from the 26 L bioreactor was transferred into the 1.3 m^3^ bioreactor under aseptic conditions, and the fungal cultivation was performed for 48 h at 35 ℃.

After 48 h, fungal biomass was harvested by filtration of the cultivation broth using brew bags. The biomass was washed with tap water and excess water was manually pressed out. Finally, the biomass was transferred into plastic bags and stored in a freezer at -18℃ until further use. To measure the biomass yield triplicate samples of biomass were dried in the oven at 70 °C for 24 h.

### Preparation of Alkali Insoluble Material (AIM)

The biomass was mixed with water to obtain a suspension with a dry weight of 1% (w/w). The water/biomass suspension was further ground in an ultrafine grinder (Masuko, Japan) with fine silicon carbide (MKE #46) grinding stones (suitable for soft materials) with an open gap of 50 μm. The suspension was ground once at the lowest speed (approximately 750 rpm) and once at 2700 rpm. The biomass was then separated by filtration. Afterwards the ground biomass was mixed with 0.2 M NaOH solution (50 mL/ 1 g dry weight biomass), stirred for 1 min, and heat treated in the autoclave for 20 min at 121℃. The alkali insoluble materials (AIM) of biomass were separated by filtration using a brew bag and washed repeatedly with water until a neutral pH was achieved.

### Fungal hydrogel preparation

The fungal hydrogel was prepared in two steps. In the first step, AIM was mixed with 5 mL of 0.25 M lactic acid / 0.1 g dry weight AIM and mixed to a rich concentration of 2 wt% (dry weight). This led to the formation of hydrogels. The final pH was regulated by adding lactic acid (0.25 M) or base (1 M NaOH) to decrease the pH (to 2) or increase the pH (to 3 or 4) respectively. The obtained hydrogel was further processed in the ultrafine grinder three or six passes with an open gap of 50 μm at a speed of 2700 rpm. Afterwards, the ground hydrogel was vacuum filtered using a vacuum funnel set up (Merck, Germany) through a nylon filter of 30 μm (Spectra/Mesh^®^, Woven Filters) to obtain 4–5 wt% concentrated hydrogels.

### Dry gel spinning of fungal hydrogel

**Fig. 1:**
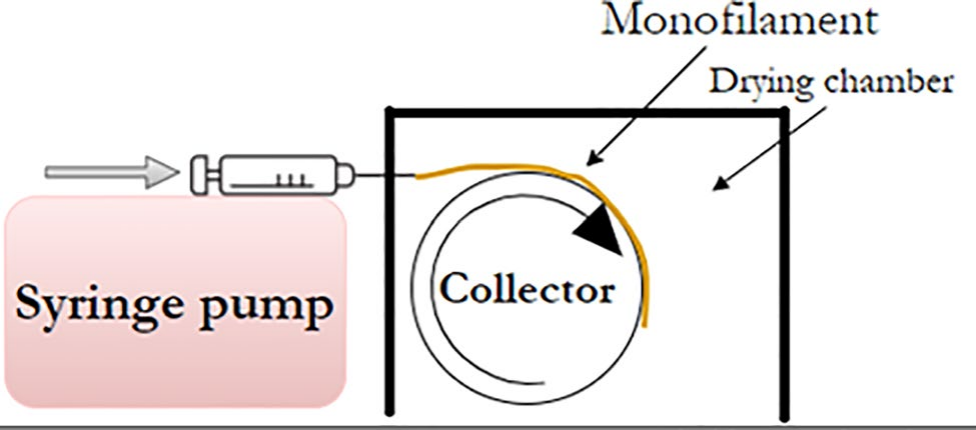
The dry gel spinning setup included a syringe pump that pressed out the fungal hydrogel through a needle on a rotating collector inside a drying chamber

The dry gel spinning process of the fungal hydrogel was developed using a small-scale custom-made setup. A syringe (10 mL) was placed in a syringe pump (World Precision Instruments, United Kingdom) such that the syringe needle was located in front of a collector wheel covered with parafilm wrap (Parafilm^®^, Bemis, USA). The collector wheel was connected to a brushless direct current (DC) motor (Oriental Motor, Model GFV4G100S, Japan). The syringe was filled with concentrated fungal hydrogel, and the hydrogel was injected through a needle with dimensions of 1.20 × 50 mm or 0.80 × 40 mm (numbers represent as, needle diameter x needle length). The speed of the pump was set to 0.5 mL/min and the collector was rotated with an adjustable speed to ensure collection of the as-spun monofilament without any damage to it. The monofilament was left on parafilm and dried at room temperature before being removed and characterized. To study the effect of the drying conditions on the filament properties, a drying chamber was built (using plexiglass sheets with a thickness of 5 mm). The collector was placed inside the chamber, whereas the syringe pump was located outside the chamber. The temperature inside the chamber was adjusted to 30 °C during dry gel spinning (Fig. 1). The abbreviations of the different monofilaments produced are explained with details in the Table 1.

**Table 1:** Abbreviations for each fungal monofilament, as well as filament diameter with respect to number of grinding passes, pH and concentration of the hydrogel, and dimension of the spinneret (needle) – diameter x length

Abbreviation for the monofilament	pH of hydrogel	Hydrogel concentration (wt%)	Grinding passes	Needle dimension (mm)	Monofilament diameter (mm)
F-pH2-1.2(3)	2	7.70	3	1.20 × 50	0.287 ± 0.015
F-pH3-1.2(3)	3	4.65	3	1.20 × 50	0.191 ± 0.004
F-pH3-1.2(6)	3	4.80	6	1.20 × 50	0.214 ± 0.007
F-pH4-1.2(3)	4	5.00	3	1.20 × 50	0.210 ± 0.037
F-pH4-1.2(6)	4	4.60	6	1.20 × 50	0.260 ± 0.020
F-pH3-0.8(3)	3	4.60	3	0.80 × 40	0.114 ± 0.013
F-pH3-1.2(3) HC^1^	3	4.79	3	1.20 × 50	0.189 ± 0.004
F-pH3-1.0(3) MC^2^	3	4.65	3	1.00 × 50	0.158 ± 0.025

^1^ The hydrogel spun while drying in a heated chamber (HC)

^2^ The hydrogel injected with the micro compounder and let out through a tube and spun through a needle directly on the rotating collector

#### Upscaling the gel spinning process with micro twin screw compounder

**Fig. 2:**
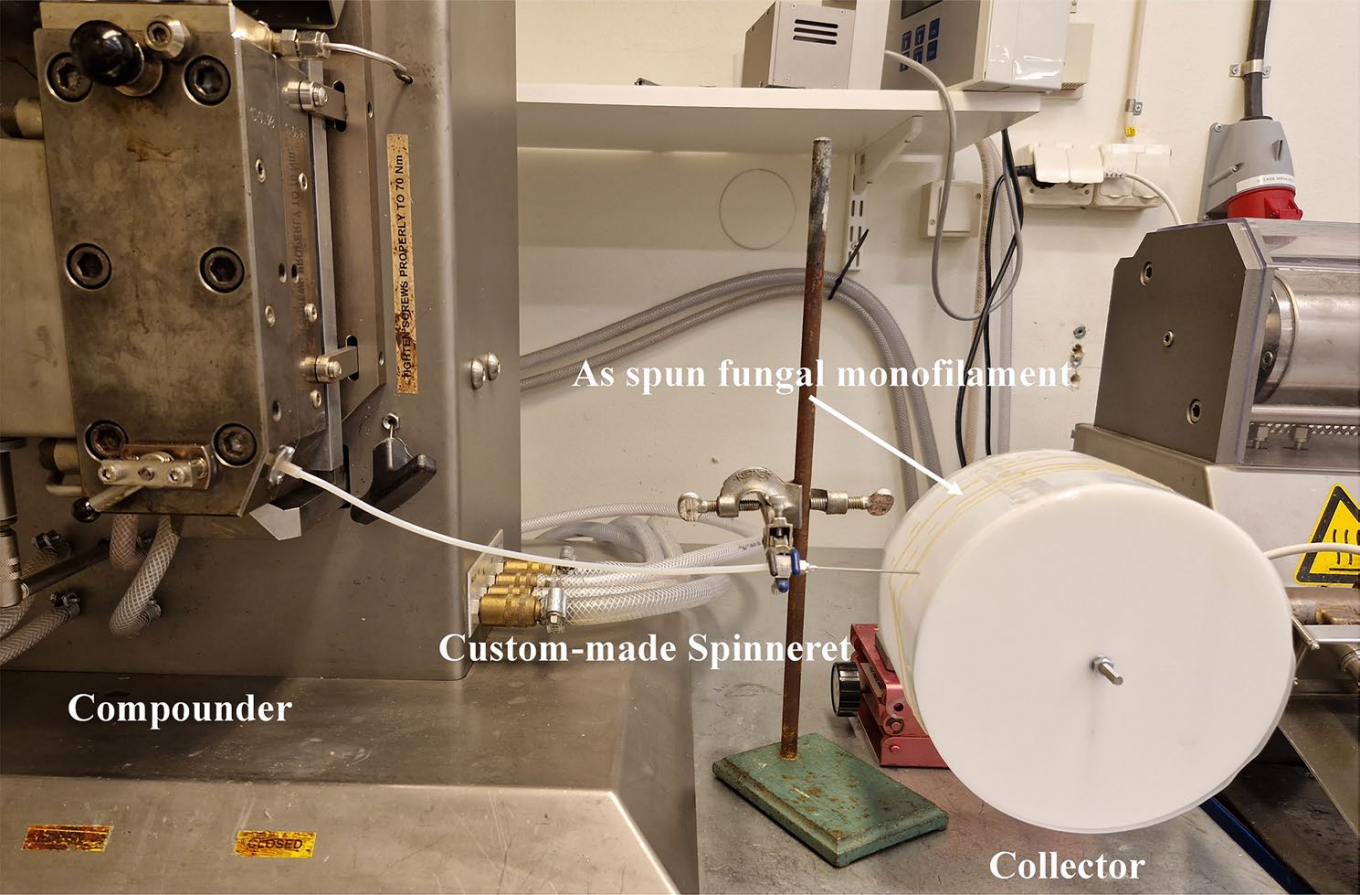
The scale-up of dry gel spinning process using a Micro 15 cc Twin Screw Compounder as a pump

The Micro 15 cc Twin Screw Compounder (Xplore Instruments, The Netherlands) was used to inject the fungal hydrogel as a replacement for the syringe pump (Fig. 2). The process was initiated by injecting 15 g of fungal hydrogel into the compounder and mixing the hydrogel inside the compounder at a speed of 50 rpm at room temperature for approximately 5 min. A custom-made spinneret, which was a tube approximately 200 mm in length, was attached to the compounder. A needle was connected at the other end of the tube, and the hydrogel was pumped from the compounder and spun through the needle. The as-spun monofilament was placed directly on a rotating collector covered with Parafilm. The speed of the twin screws was adjusted to 2 rpm during spinning.

### Twisting monofilament into a multifilament yarn

To prepare multifilament yarns, three or six filaments were placed in a hand-twisting tool (Max Kohl A.G. Chemnitz, Germany) and heated for 15 s using a steam cleaner. A twist was applied with 15–20 twists/m. The procedure ended with three heat streams of approximately 2 min to set the multifilament twist.

### Characterizations

#### Rheological testing

The oscillatory and flow rheological analysis of the hydrogel at pH 3, and concentration 4.6% was conducted using a Malvern Kinexus rheometer. The rheometer was equipped with a serrated plate–plate geometry, featuring a 40 mm diameter and a 1 mm gap distance. The temperature was maintained at 25 °C using a Peltier system and a solvent trap was employed to prevent evaporation. Prior to the analysis, the hydrogel sample underwent a 10-minute equilibration period.

The time sweep test was performed under constant strain (0.1%) and frequency (1 Hz) to ascertain the equilibrium elastic modulus of the sample within the linear viscoelastic (LVE) regime. Additionally, flow analysis was performed across a range of 0.01 to 1000 s^− 1^ for the same hydrogel using automated acquisition time mode and shear-rate control to prevent flow instabilities.

#### Scanning Electron Microscopy (SEM)

AIM and fungal hydrogels at different pH values were quickly frozen in liquid nitrogen to maintain their original morphological structure and further freeze-dried in a Labconco freeze dryer (USA) before SEM analysis. Monofilaments were cut into 2 cm pieces for analysis. Surface images were obtained using an ultra-high-resolution field-emission scanning electron microscope (FE-SEM) (Zeiss, Sigma, Germany) after they were affixed to carbon tape and coated with gold. Photomicrographs were captured at 100× magnification for freeze-dried AIM and hydrogels at 300× magnification for monofilaments (accelerating voltage of 20.00 kV).

#### Tensile testing

Tensile testing was conducted according to SS-EN ISO 527-1:2019 with a Tinius Olsen (United States) tensile machine using a load cell of 100 N, two 100 N grips, crosshead speed of 1 mm/min, pre-load of 0.01 N, and gauge length of 20 mm. Assuming the monofilament cross-section is nearly circular, the diameters were measured using an optical microscope (Nikon. Japan) and image processing software NIS Elements V 5.11.01 (Nikon, Japan). The monofilaments and multifilament yarns were cut into approximately 40 cm pieces and conditioned according to SS-EN ISO 139:2005 in a climate chamber (Memmert GmbH, Germany) at 23 ± 2 °C and 50 ± 4% relative humidity for 24 h before tensile testing. A minimum number of 5 tests per sample was carried out.

#### Linear density and tenacity

Linear density and tenacity measurements were performed using a Favimat + single fiber tester (Textechno, Germany) with standard clamps. The pretension was set to 0.2 cN for the F-pH2-1.2 (3) samples and 0.5 cN for all the others. The gauge length was 20 mm and the test speed was 5 mm/min. The linear density of the monofilaments was measured using the vibration method (according to ASTM D 1577). The monofilaments were conditioned at 23 ± 2 °C and 50 ± 4% relative humidity according to SS-EN ISO 139:2005 in a climate chamber (Memmert GmbH, Germany) for 24 h before the test.

### Statistical analysis

Statistical analysis was performed on tensile strength and elongation % at break data using the Minitab^®^ 19 Statistical Software. Analysis of variance (ANOVA) was conducted at a 95% confidence level, which was regarded as statistically significant. Error bars in graphs represent standard deviations (SD), and the data are presented as mean values ± SD. A p-value less than 0.05 was considered as an indication of a significant effect.

## Results & discussion

The textile industry has the second-largest impact on the environment after the oil industry; therefore, novel environmentally friendly textile materials are desired. Bread waste is the most common food waste in Sweden and has been employed as a resource for the development of new textile materials. Fungal biomass was obtained and treated to produce a hydrogel by growing *R. delemar* on bread waste. Subsequently, a novel dry gel spinning method was developed to spin the fungal hydrogel directly into a monofilament without the need for solvents or coagulating agents. The process was scaled up to prepare continuous mono- and multi-filaments.

### Bread pretreatment and fungal cultivation

Previous research has shown that submerged cultivation of *R. delemar* using bread waste powder suspended in water as a substrate results in good fungal growth [7]. However, the remaining undigested bread particles were attached to the fungal biomass and could not be easily removed. The presence of these particles in biomass negatively influences the properties of the final products [6]. The preliminary experiments in this work also indicated clogging of the needle in the filament spinning process owing to the presence of these particles. Therefore, in this study, bread was subjected to a pretreatment process using amylase to liquify the starch present in the bread and use only the liquified fraction of bread for fungal cultivation. A 20% suspension of bread in water was subjected to amylase pretreatment. The suspension was highly viscous at the beginning of the enzymatic pretreatment. The viscosity of the slurry decreased after 2 h (Supporting video S2 & Supplementary document figure SD1). This confirmed the progress of the starch hydrolysis and liquefaction. After 2 h, the slurry was filtered to remove insoluble particles, and the collected liquid contained 14.7 wt% solids. This was diluted to 4 wt% solids and used for fungal cultivation. The harvested biomass was free from particles and had a meat-like structure with the yield of 0.072 g biomass/g substrate. Benedikt Maria Köhnlein, Abitbol [6] have reported a slightly higher yield of 0.095 g biomass/ g substrate in their 22 h cultivation of *R. delemar* on powdered bread suspension media. In contrast Wijayarathna, Mohammadkhani [9] have reached to a twofold yield of 0.15 g biomass / g substrate after 48 h cultivation of the same fungus in the suspended bread powder media. One reason for these higher yields could be the entangled particles in the biomass that counted as biomass itself in weight measurements. Another reason could be, due to the higher nutritional value of whole bread compared to bread hydrolysate [23] the fungi received all the nourishments needed for their growth. The particle-free fungal biomass was subjected to alkali treatment to obtain the alkali-insoluble material (AIM). AIM was used to prepare the spinnable fungal hydrogels.

### Preparation of fungal hydrogels

The fungal hydrogels can be prepared from AIM by protonating the amino groups of chitosan available in AIM [16]. The obtained dilute gel was ground to improve the homogeneity of the gel. Svensson, Bucuricova [7] and Svensson, Ferreira [8] reported hydrogel formation before wet spinning by adding a higher concentration of lactic acid (1 mL 3.5 M lactic acid / 0.1 g AIM dry weight). This study used a lower concentration of lactic acid and a diluted AIM suspension that could easily pass through a grinder (Supplementary document Figure SD2). However, dry gel spinning of the diluted hydrogel was not possible as the monofilaments turned into thin films when dried. Therefore, the process was improved by vacuum filtration to increase the hydrogel concentration to 4–5 wt%. The resulting concentrated hydrogels were suitable for dry gel spinning.

#### Morphology of fungal hydrogels

**Fig. 3:**
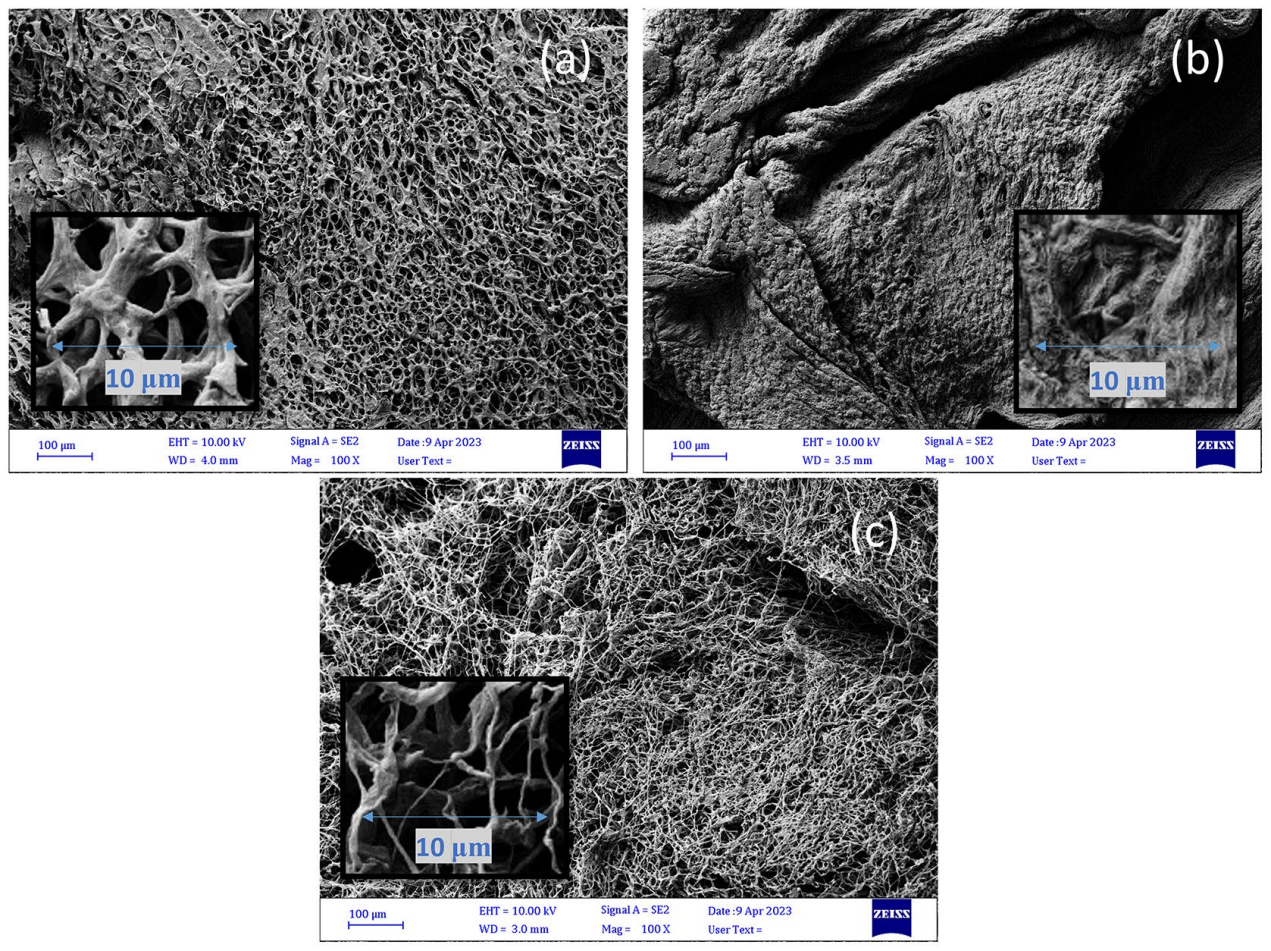
SEM images of the freeze-dried fungal hydrogel at pH 3 (4.7 wt%) (**a**) and pH 2 (7.1 wt%) (**b**), both made after three grinding passes, as well as washed AIM (2.4 wt%) (**c**). (The scale bars show 100 μm in the bigger image and 10 µmin the zoomed smaller image)

SEM analysis of the freeze-dried hydrogels indicated a porous and entangled fibrous structure for AIM and the fungal hydrogel at a pH of 3. When comparing the AIM Fig. 3c to the hydrogel at pH 3 (Fig. 3a), the entangled fibers show ‘swollenness’. This was caused by the addition of lactic acid to the AIM during hydrogel preparation, which protonated the amino groups in chitosan and resulted in gelling of the AIM [24]. In their article Cheng, Dickwella Widanage [25] have used solid state NMR to describe the cell wall organization of *R. delemar* as a mixture of densely packed crystalline domains and flexible segments of chitin and chitosan with a degree of deacetylation of approximately 47%. Due to this complex structure chitosan cannot be dissolved with acid thus when protonation of amino acid groups of chitosan takes place the fungal microfibers show swollen behavior. The swelling was also reported by Svensson, Bucuricova [7] and Svensson, Ferreira [8]. However, swelling was much higher in the hydrogel at pH 2 in such a way that the entangled fibrous structure was not visible (Fig. 3b). The entangled fibrous structure is expected to improve the formation of monofilaments and maintain their structure after drying. Lundahl, Berta [26] have also reported the importance of entanglement of the cellulose nanofibers in possibility for spinning of cellulose nanofibrils hydrogels to filaments.

#### Rheological properties of fungal hydrogels

**Fig. 4:**
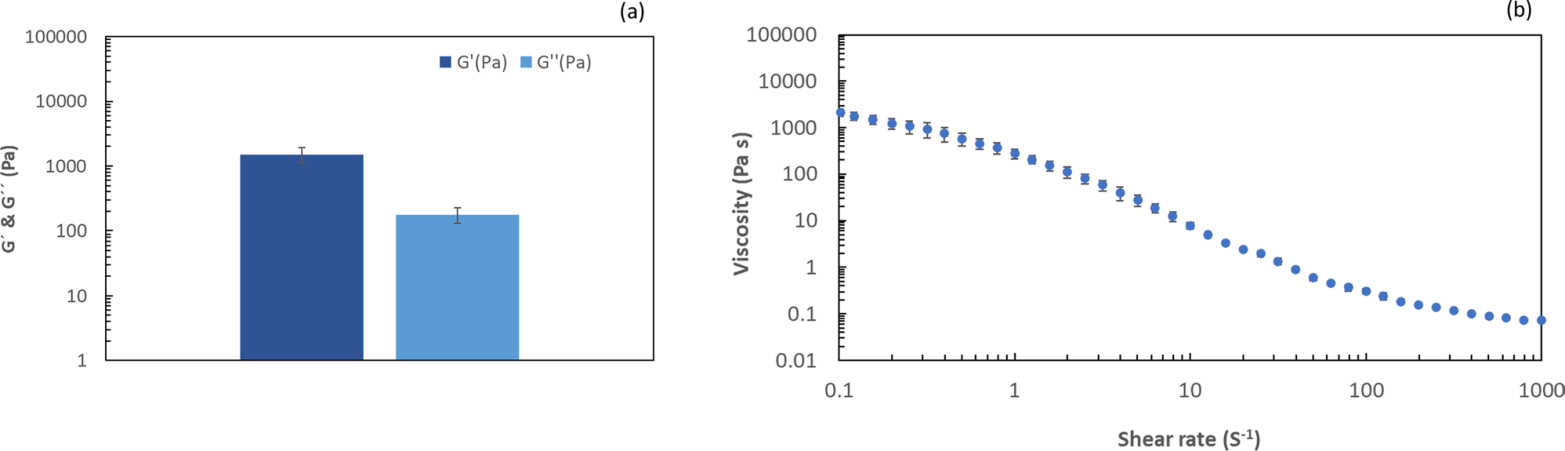
The rheological properties of fungal hydrogel (pH 3) at 4.6% concentration. In **a**), the equilibrium modulus (G′) and loss modulus (G′′) and **b**) the viscosity profile of the fungal hydrogel over a shear rate ranging from 0.1 to 1000 s^-1^ are shown. The graphs were plotted using Microsoft Excel (Microsoft^®^ Excel^®^ for Microsoft 365 MSO (Version 2402 Build 16.0.17328.20124, 64-bit)

The storage modulus (G′) and loss modulus (G′′) are important rheological parameters used to characterize biopolymer hydrogels. These moduli represent the ability of the material to store and dissipate energy. In the context of gelation, the relationship between storage and loss moduli provides insight into the mechanical properties of the gel. A fibrillar network can be observed in hydrogels, where the storage modulus (G′) was higher than the loss modulus (G′′). This is a typical behavior for gels [27]. As shown in Fig. 4a, the hydrogel at pH 3 and concentration of 4.6% exhibited viscoelastic behavior because the storage moduli were larger than the loss moduli (G′ > G′′). The storage modulus of the hydrogel was 1.50 kPa which indicated gel strength of the hydrogel. It is important to note that hydrogels with higher gel strengths are less prone to deformation and breakage during spinning, resulting in a more reliable and consistent production process.

The investigation of hydrogels encompasses an exploration of their flow characteristics and the organization of fibrillar networks induced by shear during the spinning processes. As the fibrous network underwent reorganization, there was a notable reduction in viscosity as the shear rate increased, giving rise to a beneficial shear-thinning effect. This particular flow behavior has substantial advantages for spinning processes, contributing to improved efficiency and performance. As shown in Fig. 4b, the viscosity of the fungal hydrogel (pH 3, 4.6%) decreased with increasing shear rate from 0.01 to 1000 s^− 1^. A similar observation was also noted in cellulose nanofiber hydrogels, which was attributed to the fibrillar network and fibrous entanglement within the hydrogel [24]. This suggests that fungal hydrogels exhibit behavior comparable to that of cellulose nanofiber hydrogels.

### Dry gel spinning

In preliminary experiments, it was observed that the concentration of the hydrogel plays a major role in the dry gel spinnability of fungal hydrogels. The formation of stable and continuous filaments is not possible at hydrogel concentrations below 4%. Hydrogels at higher concentrations were spinnable, and the spun monofilament was stable and flexible after drying. The hydrogels were first injected using a syringe and needle onto the surfaces of different materials that are, glass, baking paper, aluminum foil, and parafilm, to find the best material that facilitates the formation and collection of the spun monofilaments. Parafilm was identified as the optimal surface for dry gel spinning due to its ability to reduce the flattening of monofilaments during drying and facilitate easier removal of dried monofilaments. Therefore, the surface of the collecting wheel was covered with parafilm. Using the collecting wheel, which rotated and moved the as-spun monofilament away from the injecting needle, resulted in the formation of continuous monofilaments that were several meters long. The dry gel spinning process is shown in Supporting video S1 (supplementary materials).

Fungal hydrogels at different pH values (2, 3, and 4) were used for monofilament spinning after concentration via vacuum filtration. The concentration of the hydrogels was approximately 4.6-5% for the hydrogels at pH 3 and 4. In contrast, at pH 2, the hydrogel concentration was 7.7%. The lower concentration, that is, higher water content, at pH 3 and 4 compared to pH 2 was also confirmed by the more porous structure of the hydrogels at pH 3 and 4, which facilitated the entrapment of water inside the entangled fibrous structure in those hydrogels (Fig. 3a). In contrast, the non-porous and swollen structure of the hydrogel at pH 2 (Fig. 3b) enhanced water removal from the dilute hydrogel and resulted in a hydrogel at higher concentrations. In most experiments, a needle with diameter of 1.2 mm was used for spinning. After drying, the monofilaments had a diameter of 0.189–0.260 mm for the hydrogels at pH 3 and 4, whereas the monofilament spun from the hydrogel at pH 2 had a diameter of 0.287 mm. The larger diameter is related to the higher concentration of the hydrogel at pH 2.

Among the three tested pH values, the hydrogel at pH 3 (at a concentration of 4–5 wt%) was identified as the best hydrogel for dry gel spinning, in terms of the possibility of injecting the hydrogel with a uniform flow, placement of the as-spun monofilament on the collecting wheel, and stability of the spun monofilament after drying. For this pH needle with diameter of 0.8 mm was also tested, which resulted in the formation of monofilaments with a diameter of 0.114 mm. Owing to the limited capacity of the syringe pump to pump the hydrogel, smaller diameters than 0.8 mm could not be tested. The monofilaments obtained were nearly round in shape (Supplementary document Figure SD3). At pH 3, hydrogels with < 4 wt% were also tested for dry gel spinning. This resulted in the formation of flat film-like filaments.

Finally, preliminary investigations showed that for a hydrogel at pH 3, a concentration higher than 4% and parafilm resulted in the formation of a continuous monofilament. At pH4, a higher hydrogel concentration (4.5-5 wt%) was required to ensure a uniform flow in the spinning process. This is because of the lower level of protonation of the amino groups at this pH, which results in lower gelling. Spinning of the material at pH higher than 4 was not possible. Dry gel spinning of the hydrogel at pH 2 was smooth and controllable, resulting in stretchable rubbery-like monofilaments.

The formation of multifilament yarns through the twisting of monofilaments was also successful. Steaming of the monofilaments prior to twisting facilitated the twisting process, and multifilament yarns with three or six monofilaments were made with length of 10–15 m.

### Morphology of monofilament produced by dry gel spinning

**Fig. 5:**
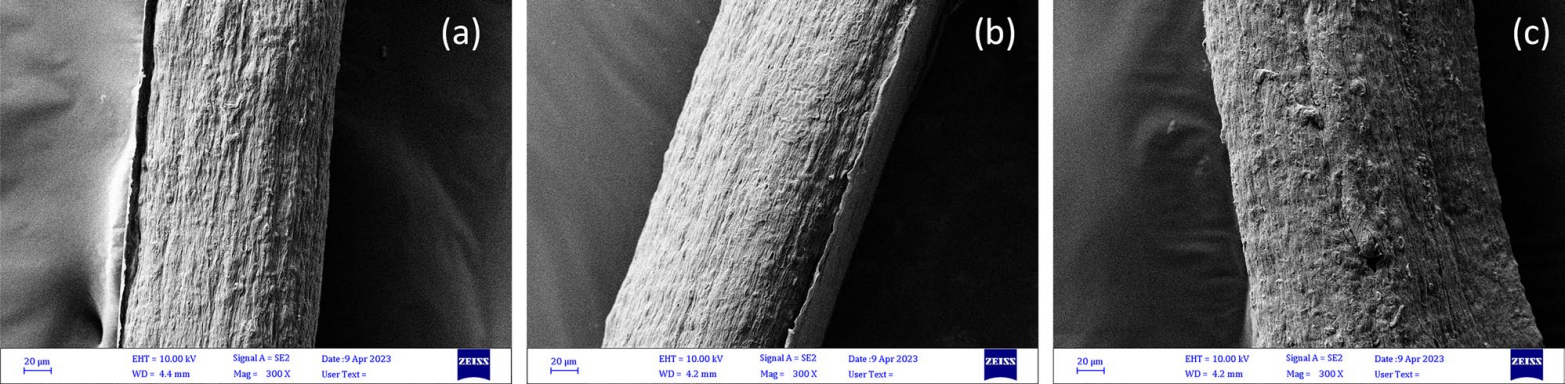
SEM images at 300×magnification of fungal monofilaments produced from hydrogels at (**a**) pH 3 with 3 grinder passes [F-pH3-1.2(3)], (**b**) pH 4 with 3 grinder passes [F-pH4-1.2(3)], and (**c**) pH 2 with 3 grinder passes [F-pH2-1.2(3)]. The scale bar shows a length of 20 μm

SEM images of monofilaments made from hydrogels at pH 3 and 4 showed a parallel pattern at the surface of the monofilaments, which indicates the orientation of fungal microfibers along the monofilament axis Fig. 5a-b during the dry gel spinning process. A similar pattern has been reported for fungal monofilaments produced by wet spinning [8, 16]. The surface of the monofilament formed from the hydrogel at pH 2 was less oriented. This could be due to the more swollen structure of the hydrogel at pH 2, which limited the possibility of orientation of fungal microfibers along the monofilament axis. A similar structure has been reported by Svensson et al. [8] for monofilaments wet-spun from a hydrogel, to which a high amount of lactic acid was added. Generally, fungal monofilaments produced by dry gel spinning showed comparable surface morphology to filaments dry-spun from nanocellulose hydrogel [20].

### Mechanical properties of fungal monofilaments produced by dry gel spinning

#### Tensile strength, elongation % at break, and elastic modulus

Tables 2 and 3 present the mechanical properties of monofilaments produced from fungal hydrogels under different conditions. In addition to the hydrogel pH, the effect of the number of grinding passes on the final properties of the monofilaments was examined. For the three grinding passes, monofilaments made of hydrogel at pH 3 showed the highest tensile strength (62.8 MPa). The tensile strength is comparable with the results reported for the wet spinning of a fungal hydrogel with a similar number of grinding passes [16]. In contrast, the elongation at break of the monofilament (14.3%) was significantly higher (p value < 0.001) than the values reported for wet-spun fungal monofilaments [16]. In the wet-spinning process, fungal hydrogels are injected into a coagulation bath containing ethanol. The presence of ethanol results in the quick dehydration of the hydrogels and results in less flexible fibers [8, 16]. In contrast, dry gel spinning results in a lower dehydration rate because dehydration occurs during monofilament drying instead of dehydration by solvent exchange during the wet spinning process. This was expected to be the main reason for the formation of more flexible monofilaments during the dry gel spinning process. The monofilaments spun from the hydrogel at pH 4 showed comparable tensile strength and elongation at break. In contrast, the monofilaments spun from the hydrogel at pH 2 showed significantly lower tensile strength (4.1 MPa) and higher flexibility (22.3%) than the other monofilaments (p value < 0.001). This might be due to the lower orientation of the fungal microfibers due to the swollen structure of this hydrogel (Fig. 3b).

**Table 2:** The mechanical properties of monofilaments and multifilament yarns prepared under different conditions, including the Ultimate tensile strength (UTS), elongation at break (ε), Young’s modulus (E), and number of replicates (n), are presented. (F – monofilaments, Y – multifilament yarns, pH2/3/4 – pH of the hydrogel, 1.2/0.8 – diameter of the spinning needle, (3/6) – number of grinder passes)

Sample	UTS ± σ (MPa)	ε ± σ (%)	E ± σ (GPa)	*n*
F-pH2-1.2(3)	4.1 ± 0.7	22.3 ± 3.8	0.02 ± 0.00	5
F-pH3-1.2(3)	62.8 ± 3.20	14.3 ± 1.8	2.04 ± 0.13	5
F-pH3-1.2(6)	37.1 ± 2.01	16.5 ± 3.5	0.86 ± 0.16	5
F-pH4-1.2(3)	57.1 ± 2.6	13.4 ± 2.1	1.88 ± 0.16	5
F-pH4-1.2(6)	31.7 ± 1.8	10.3 ± 1.3	1.08 ± 0.11	5
F-pH3-0.8(3)	72.0 ± 2.3	12.7 ± 0.7	2.18 ± 0.14	5
F-pH3-1.2(3)HC	80.5 ± 2.9	6.7 ± 2.2	3.89 ± 0.29	5
F-pH3-1.2(3)MC	103 ± 30.2	2.35 ± 0.9	5.76 ± 0.45	10
Y-pH3-1.2(3)-3^1^	43.7 ± 2.8	11.3 ± 1.2	1.18 ± 0.13	5
Y-pH3-1.2(3)-6^1^	26.2 ± 1.8	13.8 ± 2.5	0.55 ± 0.07	5

^1^ Y represents multifilament yarns

For the hydrogels at pH 3 and 4, increasing the number of grinding passes from three to six resulted in a reduction in the tensile strength of the monofilaments. This might be due to the reduction in the size of the fungal microfibers after several grinding passes, which reduced the possibility of fibrillar entanglement of the fibers and resulted in the formation of monofilaments with a lower strength. A similar phenomenon was reported by Svensson et al. for wet-spun fungal monofilaments [15].

For the hydrogel at pH 3, reducing the diameter of the needle from 1.2 to 0.8 mm, resulted in an improvement in the tensile strength by 30%, whereas the obtained monofilament still exhibited high flexibility (12.7% elongation at break). A smaller needle size resulted in formation of thinner monofilaments with more oriented microfiber structure, thereby enhancing the mechanical strength of the monofilaments. The effect of drying in a heating chamber was also investigated for the same hydrogel. When implementing a heated chamber (at 30 ℃), the tensile strength was increased from 62.8 MPa to 80.5 MPa. In contrast, the flexibility of the monofilament reduced from 14.3 to 6.7%. Drying at 30⁰C compared to room temperature probably resulted in the formation of stronger hydrogen bonds between the fungal microfibers, which improved the tensile strength while simultaneously limiting the movement of the microfibers in the structure of the monofilament and reducing the flexibility of the monofilaments.

**Fig. 6:**
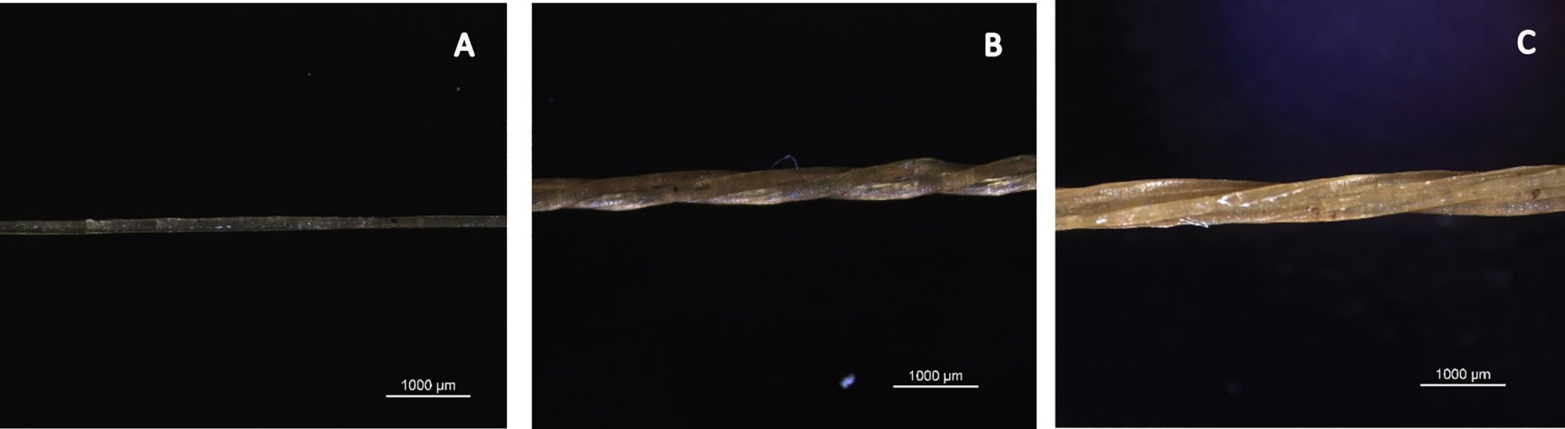
Microscopic images of single monofilament (**A**) 3 ply multifilament (**B**) and 6 ply multifilament (**C**) Yarns

Twisting of monofilaments made of the hydrogel at pH 3, could keep the monofilaments together and resulted in formation of continuous multifilament yarns (Fig. 6). The multifilament yarns had a lower tensile strength but comparable flexibility to that of the monofilaments. Multifilament yarns made of three monofilaments showed a tensile strength of 43.7 MPa, while the multifilament yarns with six monofilaments had a lower tensile strength (26.2 MPa). The reduction in strength is due to twists along the multifilament yarn length and is common for twisted multifilament yarns [28].

#### Linear density and tenacity

The linear density of the monofilaments was in the same range between 186 and 209 dtex, except for the pH2 monofilament, which had a higher linear density of 284 dtex (Table 3). Tenacity, which represents the specific strength, exhibits a similar trend in monofilaments produced with pH 2 and 3 gels with 3 and 6 grinder passes. The monofilaments from the pH 4 gel with three grinding passes showed the highest tenacity of 16 cN/tex. In the same test batch, detecting the linear density with the vibration method was difficult because of the shape variations of the monofilament cross-section. The equipment measures the linear density considering that the cross-section of the fiber is tubular. When irregularities, such as flattening and bubbles, occur, the equipment does not measure or detect linear densities with very high deviations. The tenacity of monofilaments from both pH 3 and 4 gels with three grinding passes when converted to grams per denier (g/d) are 1.11 g/d for monofilaments produced with the pH 3 hydrogel with 3 grinder passes and 1.2 mm needle (F-pH3-1.2 (3)), 1.54 g/d for monofilaments produced in the heated chamber with the pH 3 hydrogel with 3 grinder passes and 1.2 mm needle (F-pH3-1.2 (3) HC), and 1.82 g/d for monofilaments produced with the pH 4 hydrogel with 3 grinder passes and 1.2 mm needle (F-pH4-1.2 (3)). These monofilaments are comparable in tenacity with wool (1.0–1.7 g/d), Viscose (1.5–2.4 g/d), and Rayon (1.3–1.5 g/d) however, flexibility should be improved [29].

**Table 3:** Linear density and tenacity of selected monofilaments obtained from Favimat+ (F – monofilaments, Y – multifilament yarns, pH2/3/4 – pH of the hydrogel, 1.2/0.8 – diameter of the spinning needle, (3/6) – number of grinder passes)

Sample	Linear density (dtex)			Tenacity (cN/tex)			Elongation (%)			*n*
F-pH2-1.2 (3)	284.4	±	12.2	2.0	±	0.4	15.5	±	3.7	4
F-pH3-1.2 (3)	205.6	±	18.9	9.8	±	2.8	10.9	±	2.7	6
F-pH3-1.2 (6)	191.9	±	8.8	4.5	±	0.7	13.8	±	4.0	10
F-pH4-1.2 (3)	209.5	±	7.1	16.1	±	2.0	9.3	±	8.8	3
F-pH3-1.2 (3) HC	186.3	±	8.0	13.7	±	1.2	4.6	±	1.0	8

### Upscaling process with micro twin screw compounder

The dry gel spinning process was successfully scaled up with a 15 cc twin screw micro compounder as a replacement for the syringe pump. The hydrogel was properly mixed with screws. As the micro compounder showed a higher capacity for pumping the hydrogel, the use of a spinneret with a diameter smaller than 0.8 mm was possible. A 0.5 mm spinneret (connected directly to the micro compounder (without any connecting tube and needle) was used during the first trial. Spinning of the monofilament was possible using the spinneret; however, because of the low orientation of the hydrogel in the absence of the needle, the placement of the as-spun monofilament on the rotating wheel was difficult. Therefore, in the next step, a tube was connected to the micro-compounder and a 1 mm needle (1.00 × 50) was attached to the tube. Pumping the hydrogel through the tube and needle was possible, and the as-spun monofilaments were successfully placed on the surface of the collecting wheel. The dried monofilaments were continuous and exhibited a higher tensile strength (103 ± 30.2 MPa and lower elongation at break (2.35 ± 0.9%) than the monofilaments prepared using the small setup. One reason for the brittle behavior could be the higher orientation of the fungal microfibers during travel through the tube and needle. Another reason could be the mixing of the hydrogel between the two screws, which resulted in better homogeneity of the hydrogel and a higher orientation of microfibers along the monofilament. Furthermore, a similar brittle behavior has been reported in dry-spun filaments with a high orientation index made from cellulose nanofibers [20]. Nonetheless, these observations confirm that dry gel spinning of the fungal hydrogel can be easily scaled up using a pump with a higher capacity.

## Conclusions

In this article, the development of novel dry gel spinning to produce monofilaments from fungal hydrogels was successfully demonstrated. The filamentous fungus *Rhizopus delemar* was grown in hydrolyzed bread media and the process was scaled up using a pilot scale bio reactor, to obtain a particle free biomass. The alkali-insoluble material (AIM) was prepared after subjecting the biomass to an alkali treatment and spinnable viscoelastic hydrogels were produced by protonating the chitosan in AIM with lactic acid. Scaling up the dry gel spinning method with process improvements for continuous spinning and monofilament drying was achieved, along with the successful production and testing of multifilament yarns by twisting several monofilaments. The produced monofilaments show strength comparable to natural fibers like wool and regenerated cellulose fibers such as viscose and rayon. However, further research is essential to enhance the flexibility of fungal monofilaments for broader textile applications. Overall, the utilization of abundant food waste to produce biobased value-added materials, as discussed in this study, represents a noteworthy advancement towards waste valorization and mitigation of textile industry pollution.

### Electronic supplementary material

Below is the link to the electronic supplementary material.

Supplementary Material 1

Supplementary Material 2

Supplementary Material 3

## Data Availability

The raw data used in this study are available from the corresponding authors upon request.

## Abbreviations

AIM: Alkali Insoluble Material
ANOVA: Analysis of variance
