# Supplementary material for: Dry gel spinning of fungal hydrogels for the development of renewable yarns from food waste
Source: Fungal Biol Biotechnol. 2024 Aug 2;11:9. doi: 10.1186/s40694-024-00178-1 (PMC11295346; doi:10.1186/s40694-024-00178-1)
Supplement: Supplementary file 3 — Supplementary Material 3 [file 40694_2024_178_MOESM3_ESM.docx]

# Supplementary document for:

# Dry Gel Spinning of Fungal Hydrogels for the Development of Renewable Yarns from Food Waste.

Alice Lindh^1^, E. R. Kanishka B. Wijayarathna^1^*, Göksu Cinar Ciftci^2^, Samira Syed^1^, Tariq Bashir^3^, Nawar Kadi^4^, Akram Zamani^1^*

^1^Swedish Centre for Resource Recovery, University of Borås, Borås, SE-501 90 Sweden

^2^RISE Research Institutes of Sweden, Stockholm 114 28, Sweden

^3^Polymeric E-textiles, The Swedish School of Textiles, University of Borås, Borås SE-50190, Sweden

^4^Department of Textile Technology, Faculty of Textiles, Engineering and Business, University of Borås, SE-50190 Borås, Sweden

Figure SD1: Bread suspension getting treated by α-amylase. A & B – High viscous suspension at the beginning, C – Low viscous suspension when the hydrolysis takes place after ca. 1.5 h.


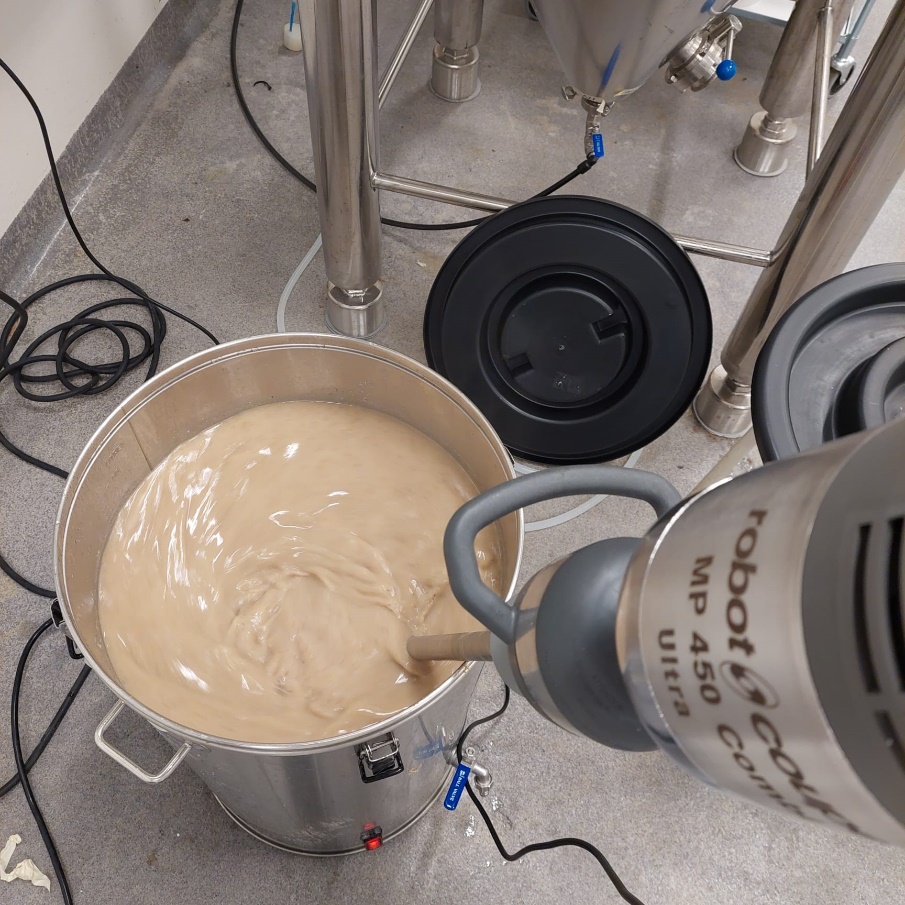

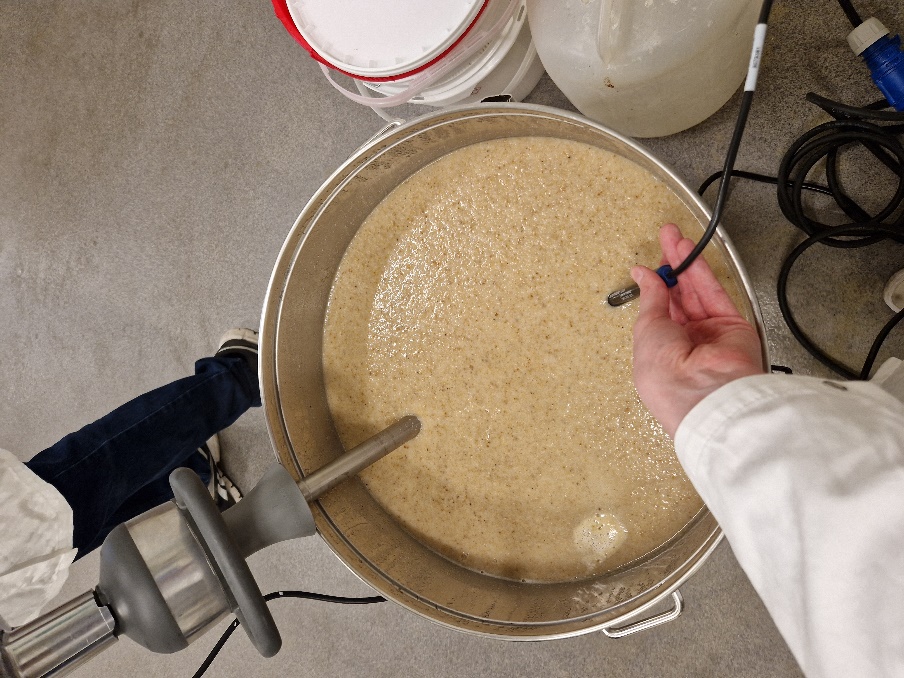

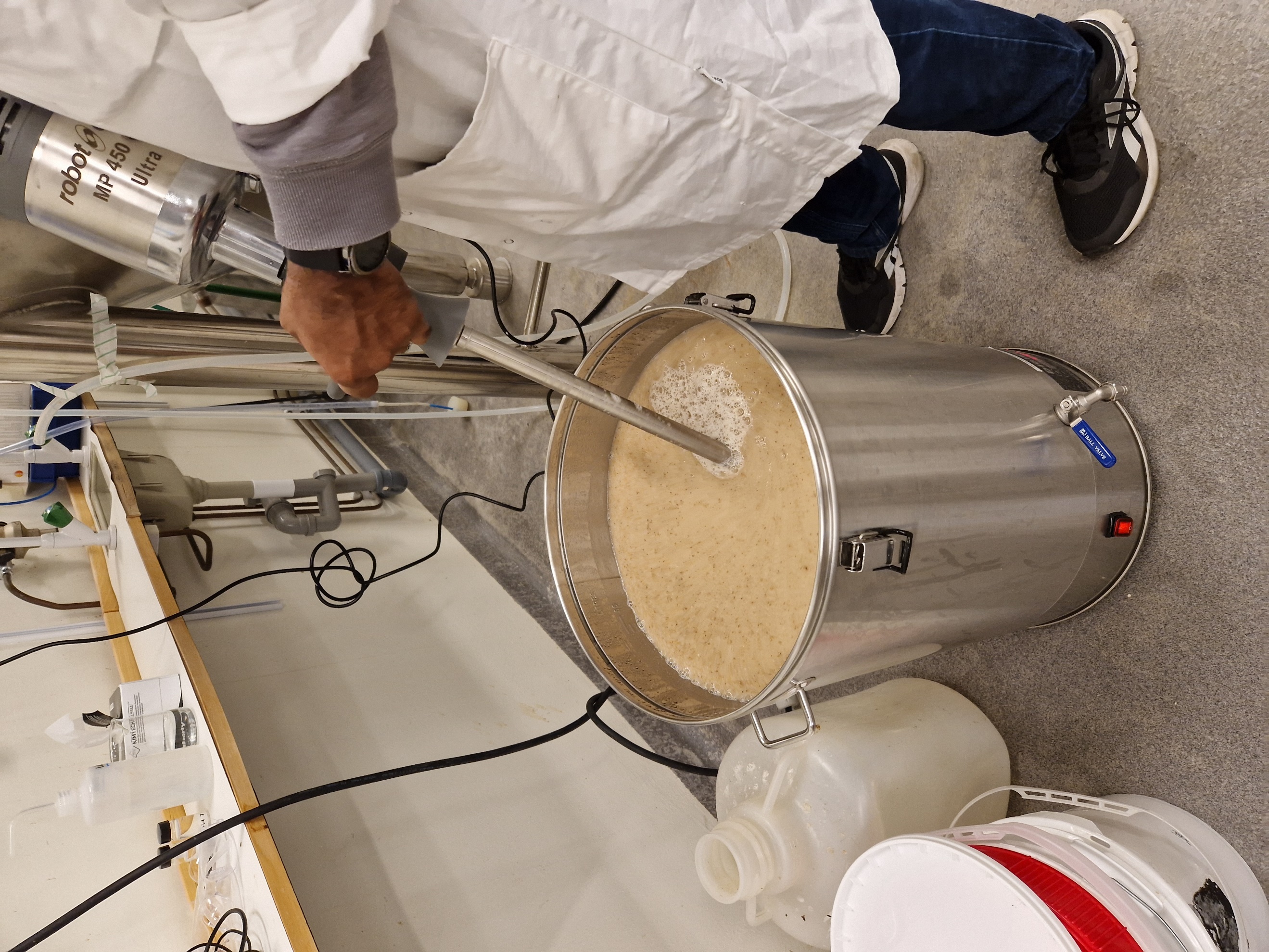


A

B

C


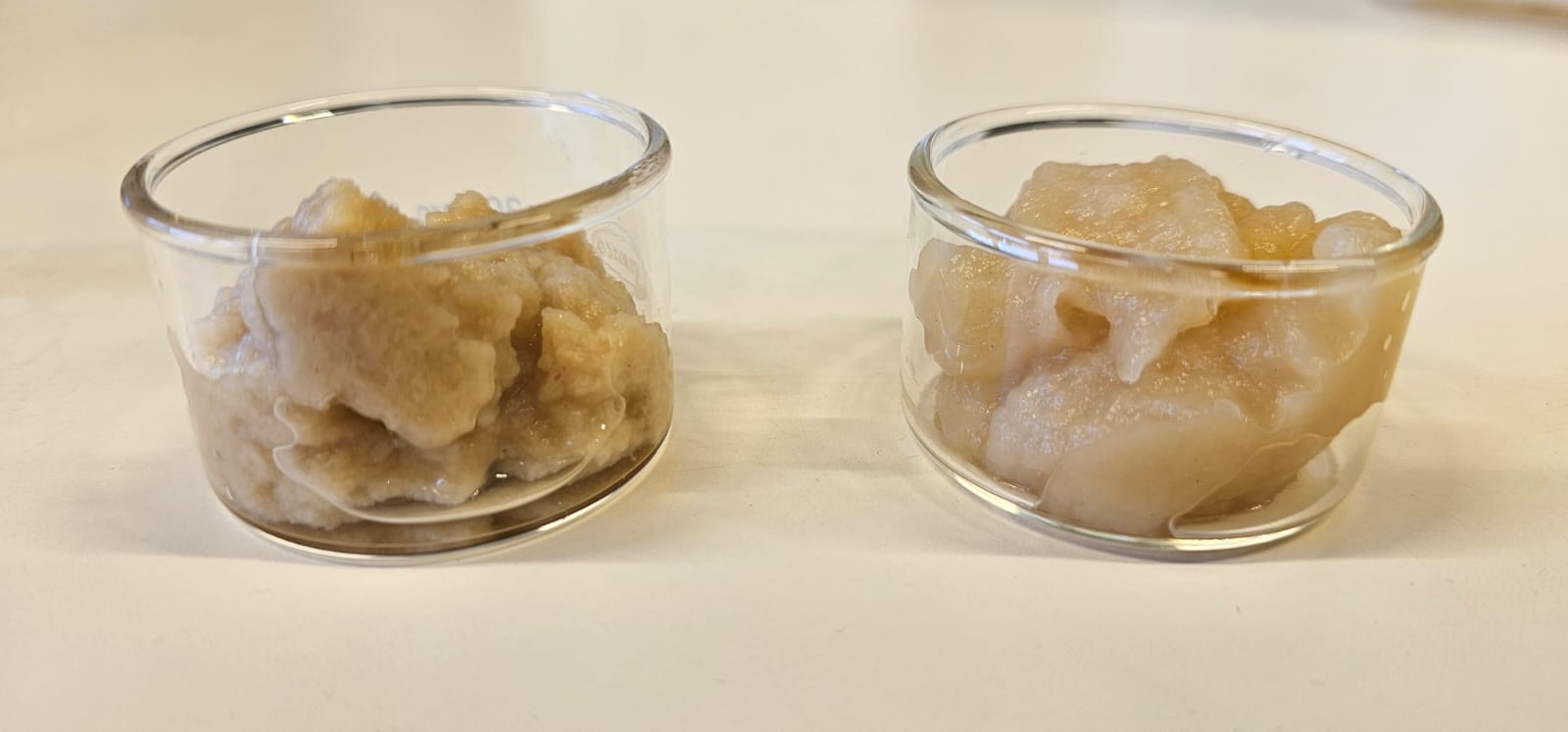


A

B


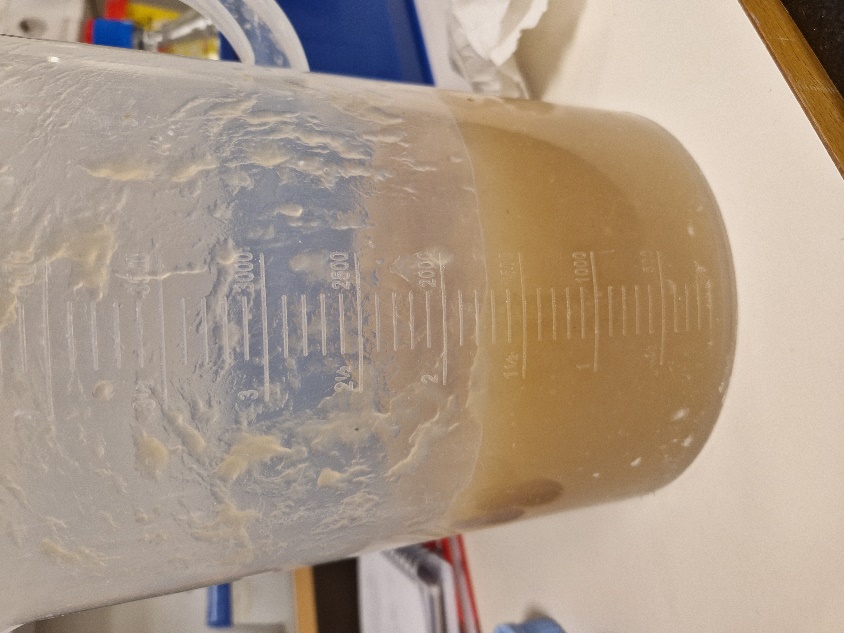

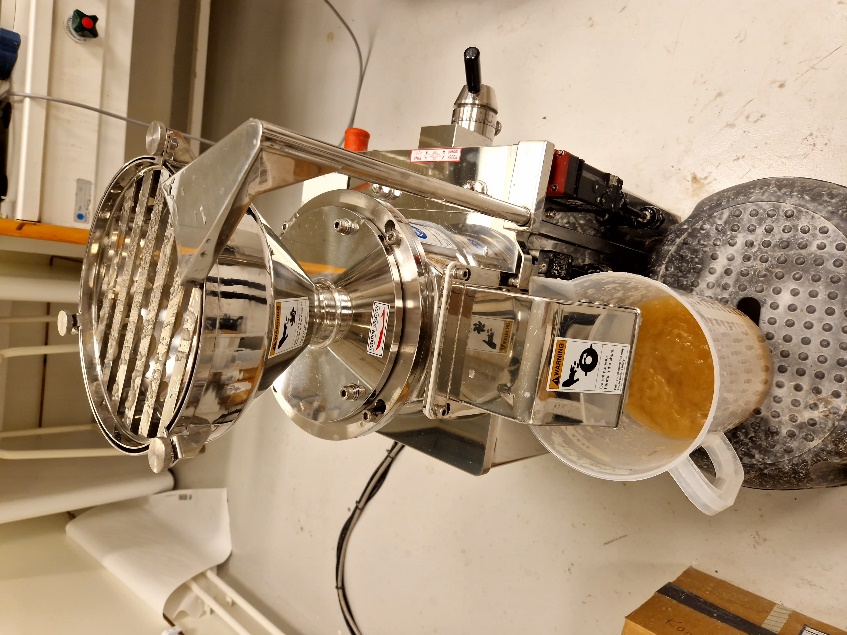


C

D

Figure SD2: A – Alkali insoluble material (AIM), B – Hydrogel of AIM after adding lactic acid, C – Diluted hydrogel before grinding, D – Hydrogel grinding using ultrafine grinder (Masuko, Japan)


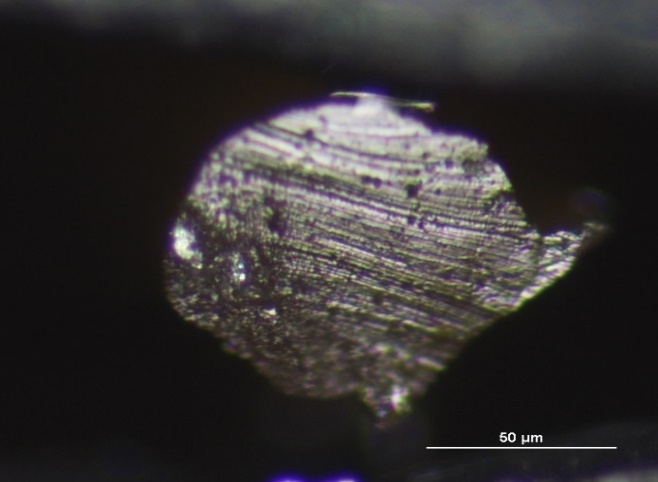

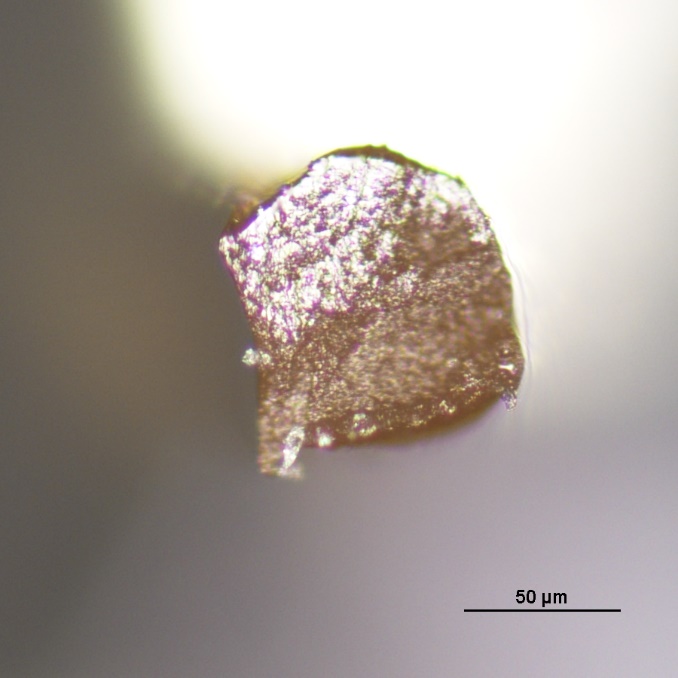

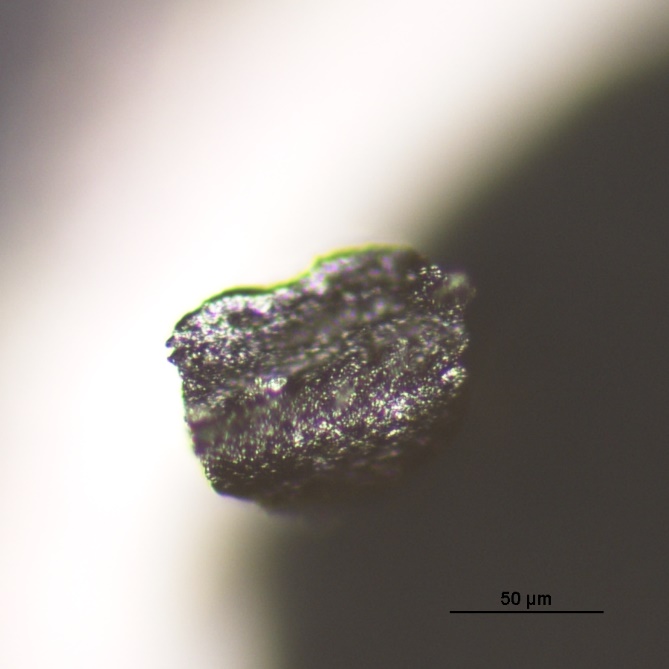

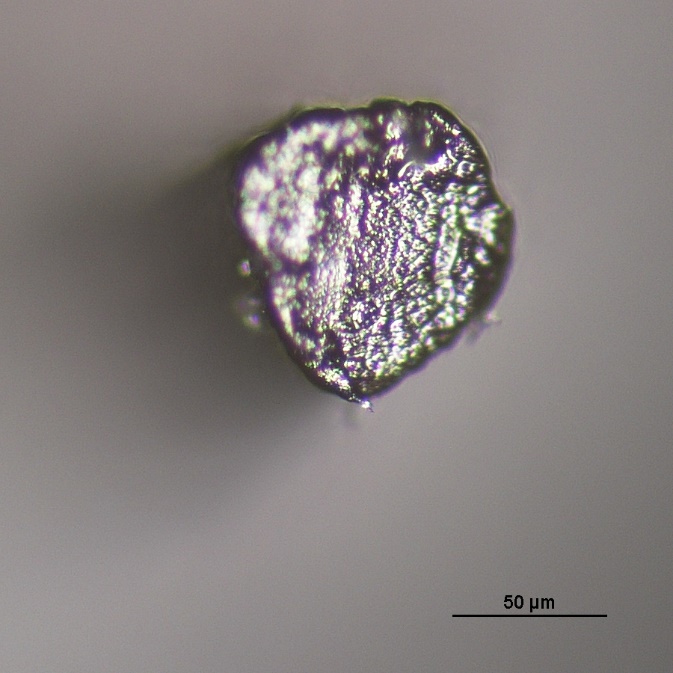


A

B

D

C

Figure SD3: Monofilament cross sections (made with pH 3, 4.6% hydrogel & 1.2 diameter needle). A, B & C – nearly circular cross sections, D – an irregular cross-section (obtained from Nikon Eclipse LV150NL - Transmission Microscope using NIS-Elements BR 5,41,01 64 bit software)
